# Farm and Livelihood Characteristics After ITM Vaccination Against East Coast Fever in Tanzania

**DOI:** 10.3389/fvets.2021.639762

**Published:** 2021-11-11

**Authors:** Nils Teufel, Luke Korir, James Hammond, Mark van Wijk, Henry Kiara

**Affiliations:** ^1^Policies, Institutions and Livelihoods, International Livestock Research Institute, Nairobi, Kenya; ^2^Sustainable Livestock Systems, International Livestock Research Institute, Nairobi, Kenya; ^3^Animal and Human Health, International Livestock Research Institute, Nairobi, Kenya

**Keywords:** vaccination, Tanzania, East Coast Fever, impacts, impact pathway

## Abstract

East Coast Fever is a critical cattle disease in East and Southern Africa which is currently mainly controlled through frequent chemical removal of ticks, the disease vector. However, a vaccine conveying life-long immunity has existed for some time, known as the infection and treatment method (ITM), although it has so far not been widely adopted because of its cost, demanding distribution system and regulatory reservations. Also, despite having proved effective on the animal level, the promoters of the vaccine have not been able to show much evidence of its benefits on the herd, farm and household levels. This study, based on a cross-sectional survey of 994 cattle keepers throughout Tanzania, aims to provide such evidence by comparing indicators of herd productivity, of farm management and success as well as of household livelihoods between households that have adopted the ITM vaccine for some years with those that have only recently adopted it. Econometric models identify the contribution of ITM adoption to indicator values together with various other determining factors amongst 277 long-term adopters of ITM and the control group of 118 recent adopters as well as 118 matched farmers without access to ITM. The results confirm that ITM adoption is positively associated with all three indicators of herd-productivity considered in this study. However, it does not support any of the three indicators of farm management and only one out of four indicators representing farm success. Nevertheless, the adoption of ITM shows a positive association with all four indicators of household livelihood. Investigating the chain of intermediate outcomes, indicators of herd productivity, such as milk yield, are significantly linked to higher feed expenses, contributing to increased livestock productivity and ultimately income and food availability. Overall, these results therefore support the promotion of ITM as a beneficial technology for the sustainable development of rural livestock keepers.

## Introduction

East Coast Fever (ECF), caused by the haemoprotozoan parasite Theileria parva and transmitted by ticks, causes considerable economic losses in 11 countries in Eastern, Southern and Central Africa. With about half of this region's 75 million cattle being at risk of ECF (1), losses caused by this disease are considerable, but quantitative assessments vary widely. For instance, in Tanzania the estimates of annual production losses due to ECF range from US$ 43 million [(2), cited by Ref. (3)] to US$ 248 million (4). The disease causes high mortality (>80%) and affects high-grade dairy cattle (5) as well as young zebu cattle in pastoral production systems (3). Pastoralists are forced to avoid areas of high ECF risk, which is becoming increasingly difficult as the ticks and infected cattle move into new areas, driven by increasing land pressure, further spreading the disease (6). Current control measures involve the use of acaricides to prevent tick infestations in up to half-weekly intervals. However, even in areas where control measures are common, such as in smallholder dairy systems in the Dar-es-Salaam region of Tanzania, ECF prevalence rates of 45% and case fatality rates of 64% have been recorded (7). Besides these risks, an acaricide-based approach to ECF control implies considerable costs and negative environmental effects, calling into question the efficacy of this approach (8). Furthermore, after prolonged use of acaricides, ticks develop resistance to the chemicals. Effective drugs for the treatment of ECF are available but they require to be used at an early stage of the disease and are often too costly for poor livestock keepers, especially for the treatment of less valuable zebu cattle. Due to the ECF risks and the associated cost of controlling the disease, many smallholders across East Africa are reluctant to adopt improved breeds of cattle, as the disease affects Bos taurus breeds more severely than Bos indicus breeds (9), an effect common to many commercializing smallholder farming systems (10).

To find a more cost-effective control of ECF, an alternative approach, the infection and treatment method (ITM), was developed more than 40 years ago. Scientists from the East African Veterinary Research Organization (now the Kenya Agricultural and Livestock Research Organization), in collaboration with international partners had first reported life-long immunization against ECF by infecting and simultaneously treating cattle with a long-acting antibiotic in the mid-70s (11, 12). During these early stages of vaccine development, concerns among scientists, policy makers and veterinary authorities about the merits of the vaccine as well as a supply driven approach to vaccine distribution had limited the dissemination and adoption of ITM. The initial concerns were mainly based on the complexity of stabilate production, the widespread field use of over-the-counter antibiotics and the potential further transmission of the disease through ticks after vaccination with live pathogens (9, 13). Despite these reservations, ITM trials proceeded, improving and standardizing the vaccine (9) and demonstrating high rates of efficacy, above 95% in some cases (3, 14). Yet despite improved understanding of the pathogen und the vaccination-induced immune response (15), obstacles to wide-spread dissemination remained. These included the characteristics of the approach [animals are infected with live parasites of varying genetic identities (16)], distribution constraints (the vaccine requires liquid nitrogen storage), vaccination costs (US$ 8–12 per animal, including a dose of a long-acting specific antibiotic) and post-vaccination reactions (depending on vaccine and treatment doses some vaccinated animals show severe ECF infection symptoms). In addition, interests in the sale of acaricides have also affected the promotion of ITM (13). This resulted in lower than expected uptake during the first two decades of the vaccine's production (14).

To achieve the greatest benefits, the vaccination is mainly targeted at calves, thereby maximizing protection throughout an animal's life and reducing the amount of required antibiotics (9). In pastoral systems this was shown to decrease mortality by more than 90% (17), resulting in increased off-take of animals and more diversified investments by pastoralists. In addition, the same study reports that vaccinated animals, identified by their ear-tags, fetched higher prices at cattle markets. Furthermore, a trend toward improved cattle breeds has been reported where ITM has been adopted in extensive systems (18). In intensifying dairy systems, the use of the ITM vaccine allows farmers to quickly reduce the frequency of tick control (from weekly or twice weekly dipping/spraying regimes to once in 2 or 4 weeks, which is still required to control other tick-borne diseases) without any detrimental animal health effects (32), cited in Refs. (11, 14)]. The resulting reduction in production costs seems to be the main direct benefit in these systems. In addition, considerable gender differences have been detected, indicating significantly higher adoption rates within male-headed households (19).

Despite the high cost of ITM compared to other vaccines (3), it has been shown that controlling ECF with the ITM vaccine results in only about 60% of herd-level costs compared to treating clinically infected calves, without the consideration of subsequent tick-control activities. Kivaria et al. (8) report a 40–68% reduction in the annual cost of controlling ECF, depending on the post immunization dipping strategy adopted. The benefits of reducing acaricide use for tick control were also determined by Lynen et al. (14). Highlighting this aspect of private profitability, it has been proposed that strengthening the role of private sector animal health services within ITM distribution systems would be a more efficient approach to achieve wider adoption of the vaccine (9).

The current distribution model of the vaccine in Tanzania is composed of multiple actors. Mandated by the African Union (AU), the Center for Ticks and Tick-Borne Diseases (CTTBD) produces the ITM vaccine in Malawi for East and Southern Africa. Within Tanzania, the Director of Veterinary Services monitors and regulates the importation of the vaccine. There are currently four private companies who are licensed to import and distribute the vaccine; these are: PharmaVacs Ltd., Vetlife consultants Ltd., Dulle Veterinary Center and Ronheam International Ltd. These distributors sell the vaccine to trained vaccinators who are licensed by the Tanzania Veterinary Association and monitored by District Veterinary Officers. The vaccinators are engaged by farmers to vaccinate their cattle.

Most studies have limited the assessment of effects and costs of adopting ITM vaccination to the animal level (20, 21). Effects on herd productivity have hardly been determined (17). A simulation study of two smallholder farms in Kenya showed the positive economic effect of ITM on whole-farm economics, but only as an ex-ante assessment (22). Following a call for more poverty-oriented research into livestock diseases (23), a recent impact assessment study shows positive relationships between the adoption of ITM and milk yield, ECF mortality and various household development indicators; however, without quantifying the intermediate farm-level effects and controlling for differences between households with various degrees of ITM adoption through an instrumental variable approach (24). While determining the effects of ITM vaccination on livelihood indicators is critical for assessing the value of this technology in contributing toward ultimate development objectives, this study also aims to better understand the pathways leading to these effects and which conditions are required to achieve them.

Accordingly, this study aims to:

- Assess how the adoption of ITM contributes to herd-productivity effects
- Determine how changes to farm management and success are linked to the adoption of ITM vaccination
- Identify differences in household-level livelihood indicators between long-term and recent adopters of ITM

## Materials and Methods

### Conceptual Framework

**Figure 1:**
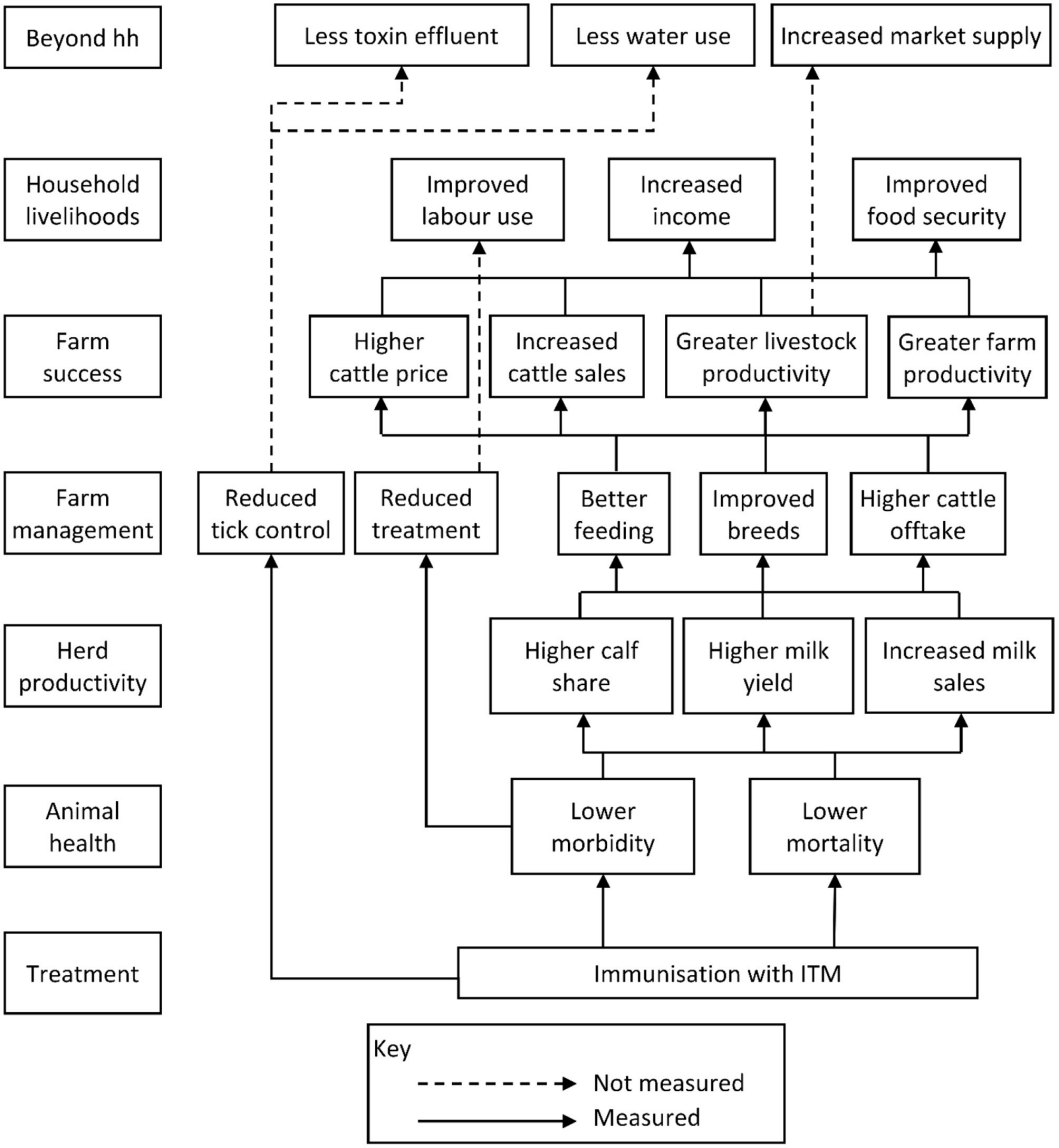
The impact pathways of ITM vaccination and their consideration in this study.

ITM vaccination is assumed to directly affect herd productivity by reducing mortality and increasing milk production. Based on these effects, ITM vaccination is expected to stimulate improvements in farm management, further intensifying livestock production, for instance though advances in feeding and breeding practices. These are expected to lead to greater farm success, as measured for instance by the rate of cattle off-take and the average revenue of cattle sales. Greater farm success then allows for the improvement in household livelihood indicators for households with a strong dependency on livestock production. Such indicators include measures of income, poverty risk or food security. Figure 1 presents the conceptual framework of the study, illustrating these pathways.

We aim to better understand the effects of ITM on farm households which have adopted this technology through the comparison of those households which have been applying the ITM vaccine to their cattle for a considerable time (the treatment group) to those which have not (the control group). To avoid self-selection bias, as would be the case when comparing vaccine adopters with those who have decided not to adopt, the control group is formed by farmers who have only recently decided to adopt the ITM vaccine, without the vaccine having yet been able to affect the health and productivity of their livestock. Because the number of farmers who had just started applying ITM was limited within the sample, additional control farmers were identified from those who had not had access to the ITM vaccine but showed similar characteristics to the recent adopters. This approach ensures a minimal selection bias as the farmers in both groups have voluntarily decided to adopt ITM, or have characteristics similar to adopters but have not had access to the vaccine.

Apart from determining the direct associations of ITM vaccination with various indicator variables, we also aim to better understand the pathways leading to these effects and the conditions required to achieve them, as illustrated in the conceptual framework. Therefore, we also investigate the links of intermediate outcomes with higher-level indicators.

### Data

The data for this study were collected through a single-round survey of livestock farmers in Tanzania. The data were collected during August/September and November 2017 by a team of trained enumerators. The data collection tool was based on the RHoMIS instrument (26), extended to cover animal health interventions and herd dynamics in greater detail and implemented in ODK.

The selection of survey respondents was based on the contacts established with 331 ITM vaccinators and other animal health service (AHS) providers from all over the country through an ILRI-led ITM dissemination project. These service providers were asked to list the number of farmers they were serving in each of the following eight categories: long-term ITM farmers (i.e., ≥2 years of ITM adoption), just-starting ITM farmers (i.e., ≤ 12 months of ITM adoption), farmers not adopting ITM with ITM vaccinators and non-adopting farmers with non-ITM AHS providers, for both pastoralists and smallholder dairy producers. For each of these categories, 24 associated AHS providers (12 for non-adopters with ITM vaccinators) were randomly selected, emphasizing providers associated with fewer categories, thereby increasing the number of providers included in the study, and those operating in regions with at least three providers, thereby focusing on areas with greater density of cattle and livestock services. This resulted in a total of 118 AHS providers being randomly selected. These were then requested to list the contacts of 15 farmers per farmer category they were associated with, resulting in a sampling frame of 2,410 farmers. From this, six farmers per type and AHS provider were randomly selected for the survey. However, the initial approach of categorizing farmers into pastoralist and dairy production systems could not be pursued because only few farmers identified themselves as pastoralists.

**Figure 2:**
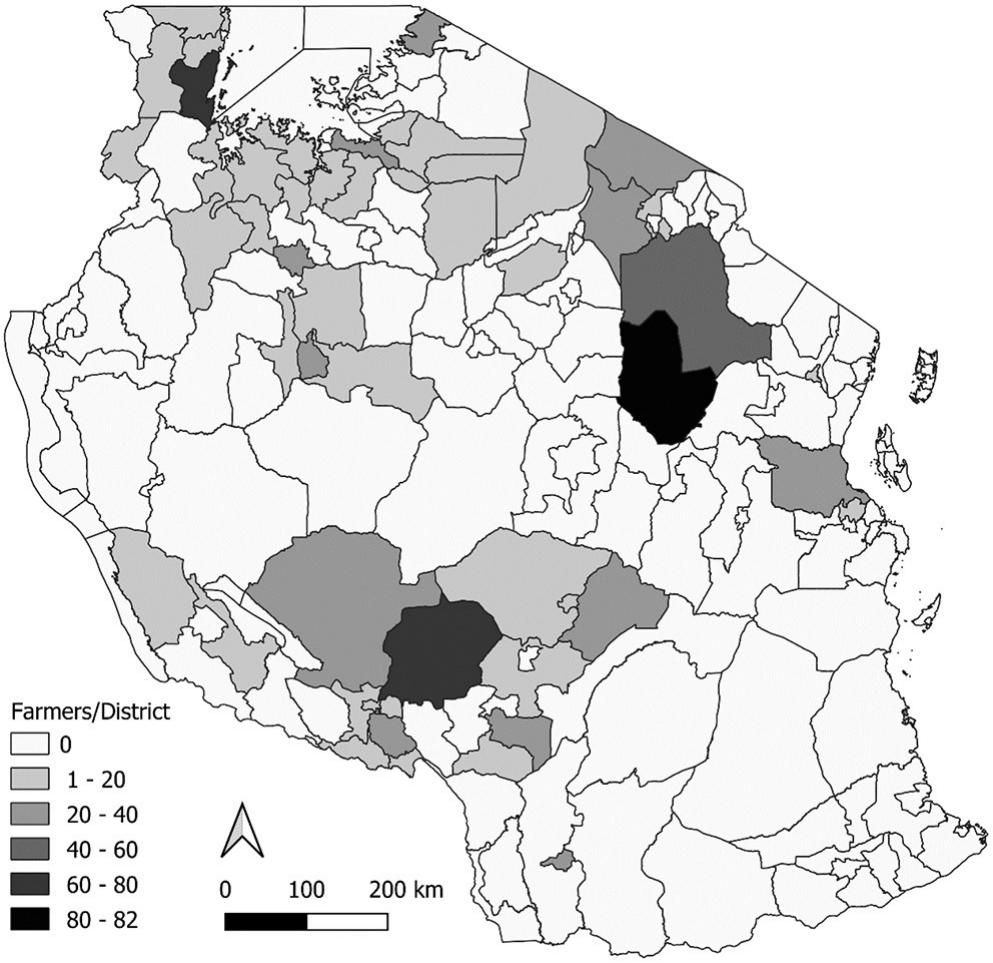
Distribution of interviewed farmers within Tanzania.

In total, 994 farmers linked to 106 AHS providers were interviewed from across Tanzania (see Figure 2), including 277 long-term ITM adopters and 119 farmers that had just started vaccinating their cattle with ITM. The final survey sample also contained 325 farmers connected to AHS providers that did not offer ITM (see Table 1). Because the number of recent ITM adopters was far smaller than planned we applied propensity score matching (PSM) to additionally select similar farmers from the non-ITM AHS providers. This approach estimates propensity scores of group membership by logistic regression. These scores were then used to match the most similar farmers of the non-ITM farmers to the just-starting farmers. The variables included in the matching process were household size, age, gender and education level of household head, herd size per household member, cultivated land, enclosed grassland per cattle unit, feed expenses per cattle unit, market orientation and proportion of off-farm income. With this we were able to match 118 farmers from the inactive areas with the 118 recent adopters included in the analysis, resulting in a control group size of 236 farmers.

**Table 1:** Survey data structure before and after propensity score matching.

**Vaccinator ITM status**	**Farmer ITM status**	**Farmers interviewed**	**Farmers included after PSM^a^**
Active	Long-term	277	277
Active	Just-started	119	118
Active	Inactive	273	0
Inactive	Inactive	325	118

a *PSM, Propensity Score Matching*.

### Methods

The research objectives mentioned above are achieved by econometric analysis of the farm-household data. Investigating the sub-samples of long-term and recent adopters of ITM, plus non-adopters matched with recent adopters by PSM, allows for the determination of the average effect of ITM vaccination on the treated (ATT) (25). The econometric analyses are based on selected indicator variables (i.e., dependent) which are then regressed on a selection of determinant variables (i.e., explanatory or independent). The indicators cover the domains herd productivity, farm management and success as well as household livelihoods, according to the conceptual framework introduced above. Herd productivity indicators include share of calves within cattle herds [calves per herd size, both measured in tropical livestock units (TLU)], milk yield (average daily milk production per herd TLU) and milk sales (milk sold per year and herd TLU). The indicators of farm management and success considered in this study include livestock management practices such as feed expenses (annual cattle feed purchases per herd TLU), keeping improved breeds (dummy variable characterizing main cattle breed type, with the responses “improved” and “mixed” categorized as “improved,” contrasting with “local”) and off-take rate (animals sold per herd size in animal numbers). The farm success indicators such as cattle price (annual cattle sales income per sold animal), average cattle sales revenue (annual cattle sales income per herd TLU), livestock productivity (value of all livestock products per herd TLU) and farm productivity (value of all crop and livestock products per area of cultivated land). Indicators linked to changes in animal health practices, such as treatment costs or acaricide control, could not be included in these analyses despite featuring in the conceptual framework because of the low number of responses to questions on these topics. Lastly, a collection of standardized indicators characterizes household livelihoods. These include gross per capita income, based on the total value of production and off-farm income per male adult equivalent (26), the Poverty Probability Index (PPI) (27), the Food Availability Index (26) and the Household Diet Diversity Score (28). The PPI, through a set of 10 questions customized for individual countries, generates a score value with which the probability of an individual household falling under a poverty line can be estimated. Within this study we use only the score value as a measure of poverty risk without actually calculating risk values. The Food Availability Index determines the calorific value of all farm products as well as of off-farm income (converted into food staples) per male adult equivalent. Finally, the Household Diet Diversity Score represents the number of food groups consumed by the household at least several times during the last week, out of a total of 10 food groups, based on the evidence that diet diversity is a robust indicator of diet quality and risk of malnutrition. Diet diversity data were collected for food scarce and food abundant seasons.

Apart from the treatment with the ITM vaccine, the econometric models also consider other variables expected to affect the indicator variables presented above. These cover various household, farm and herd characteristics and are listed below amongst the model details.

While the conceptual framework does not show two-way or feed-back relationships between practices and outcomes, it is obvious that these exist and that they may be critical in some cases. For instance, while the framework highlights the effects of productivity changes on behavior change in farm management, management practices clearly determine livestock productivity. The models consider this, by including management practices as determinants of herd productivity.

In addition to directly considering the ITM vaccination at several levels of analysis, a second set of econometric models investigates the intermediate outcomes along the impact pathway presented in the conceptual framework. To determine their contribution, they are included as determinants in the econometric models of the next level along the pathway. Accordingly, for this set of models, the direct ITM treatment variable is omitted to avoid overdetermination.

Initially, differences between treatment and control groups in outcome indicators and determinant variables are explored through independent sample *t*-tests of mean differences and chi-squared tests of association. The variation of the variables included in the results highlights the scope of further analysis. Subsequently, the association of ITM with various indicators is investigated by econometric analysis at the four levels of investigation: herd productivity, farm management, farm success and household livelihoods. All models apply ordinary least squares regression, except for the models determining the types of cattle breeds being kept, which are implemented with logistic regression due to the binary nature of the dependent variable. To avoid undue influence of exceptional observations on the results, the regression outputs were screened for influential observations, defined as being both outliers and having high leverage. Subsequently, three records were excluded from the herd and farm management level models and 6 records were excluded from the farm success and livelihood models.

### Direct Investigation of ITM Effects

#### Modeling Effects on Herd Productivity

Herd productivity is characterized by three indicators: calf share in herd [%], daily milk yield (l/TLU) and annual milk sales (Tanzanian shillings (TZS) ‘000'/TLU). The association of ITM with herd productivity is estimated by the following empirical model:


(1) $$\begin{array}{r} Y_{h}=f\left(T_{h},X_{h}\right) \end{array}$$

Where subscript *h* denotes household. *Y* represents the set of dependent variables measuring herd productivity. The variable *T* reprents a treatment dummy (0 = control, 1 = treatment). Vector *X* is composed of independent variables that control for farm and household characteristics. These are household size (number of household members), age of household head (years), education of household head (0 = primary school level and lower, 1 = post-primary), gender of household head (0 = female, 1 = male), cattle herd size (TLU/household member), area of enclosed pasture (ha/TLU), annual feed expenses (TZS ‘000/TLU), cattle breed type (0 = local, 1 = improved), market orientation (% produce sold) and off-farm income share (% of total income). Interaction terms of ITM with herd size and market orientation are also included. Within all econometric models presented here, nearly all variables, both dependent and independent, are transformed to their natural log values, increasing the models' explanatory power.

Feed expenditure and breed-type have direct short-term effects on milk yield and are therefore considered as independent factors at the herd level. On the other hand, we expect management decisions on feed and breed to be influenced by herd-level productivity in the longer-term, as illustrated in the conceptual framework. Therefore, these variables are also included as dependent variables at the farm management level.

#### Modeling Effects on Farm Management and Success

Three variables are used as indicators of farm management practices: annual expenditure on feed (TZS ‘1000/TLU), main cattle breed type (0 = local, 1 = improved) and annual off-take rate (% animals sold). At the next level, four indicators characterize farm success: cattle price (TZS ‘000/sold animals), annual cattle sales revenue (TZS ‘000/herd TLU), livestock productivity ($/herd TLU) and farm productivity ($/ha). The production values are expressed in international $ converted by purchasing power parity (PPP). The links of ITM with farm management and success are estimated using the following empirical model:


(2) $$\begin{array}{r} P_{h}=f\left(T_{h},X_{h}\right) \end{array}$$

Here the subscript *h* denotes household and *P* represents the set of farm management and success indicator variables. Variable *T* reprents the ITM treatment dummy (0 = control, 1 = treatment) and *X* is a vector of independent variables that control for farm and household characteristics as defined for Equation 1 as well as interaction terms of ITM with herd size and market orientation.

#### Modeling Association With Livelihood Indicators

A total of four indicators are used as dependent variables for modeling household livelihood outcomes. These are: annual income per capita (int. PPP $/cap), poverty probability score (PPI score), daily food availability per male adult equivalent (kCal/MAE) and household diet diversity score (HDDS) in the food scarce season. The following empirical model was used to estimate the association of ITM with each of these indicators:


(3) $$\begin{array}{r} L_{h}=f\left(T_{h},X_{h}\right) \end{array}$$

Again, the subscript *h* denotes household, while *L* represents the set of livelihood indicator variables. Variable *T* represents the ITM treatment dummy (0 = control, 1 = treatment) and *X* is a vector of independent variables that control for household and farm characteristics as defined for Equation 1 and as well as several interaction terms.

### The Contribution of Intermediate Outcomes

In an alternative approach to assessing the benefits of ITM we investigate the contribution of intermediate outcomes within the subsequent level of econometric modeling. These models have the same basic structure as the models on direct determination of ITM effects described above. However, instead of including the ITM treatment variable at each level, outcome variables of the previous level are included as determinants. Thus, the models investigating farm management practices contain the herd-productivity outcome variables: calf share, milk yield and milk sales. Similarly, the farm success models incorporate the farm management variables: feed expenses, breed type and off-take rate. Finally, the household livelihood estimations consider cattle sales price and revenue as well as livestock and farm productivity, the indicators of farm success. Interactions are not considered in these models.

## Results

The main categorization of survey respondents in view of assessing the ITM vaccination was by the year they started vaccinating. Among the 277 respondents categorized as long-term adopters, the earliest adoption was in 1998. However, the median year of adoption was 2014 and latest adoption was in 2015. Among the 118 just-starting adopters, all adoption had taken place between 2016 and 2017. Within the sample, most farmers had not vaccinated all their animals during the survey recall period of 12 months. Among those farmers for which data were available, long-term adopters had vaccinated 30% of their animals in the previous year (*n* = 232), while those who had just started had vaccinated 27% (*n* = 100).

### Descriptive Statistics

To gain some insight into the distribution of variables considered in this analysis and into differences between the main categories we compare the treatment group (long-term ITM farmers) to the control group (just-starting farmers and matched non-ITM farmers). Table 2 presents this comparison regarding variables which are expected to be dependent on the adoption of ITM, arranged by herd, farm and household levels. Results indicate that farmers in the treatment group on average showed a lower expenditure on animal feed and were less likely to keep improved breeds compared to the farmers in the control group. They also had a higher PPI score, appearing to be in greater danger of falling into poverty. The differences in means of other variables were not significant, either because the differences were small (e.g., cattle sales price) or because of large standard errors (e.g., food availability).

**Table 2:** Descriptive statistics of dependent variables for ITM treatment and control groups.

	**Control** ***n*** **=** **236**		**Treatment** ***n*** **=** **277**		
**Variable**	**Mean**	**SE**	**Mean**	**SE**	***p*-value**
Calf share [%]	7.99	0.70	9.35	0.65	0.16
Daily milk yield [l/TLU]	1.51	0.15	1.24	0.11	0.14
Annual milk sales [l/TLU]	391.70	41.29	336.68	32.44	0.30
Annual feed expenses [TZS '000/TLU]	56.89	5.74	32.32	4.05	0.00
Cattle breed type (improved = 1) [%]	63.14	3.15	46.21	3.00	0.00
Annual off-take [%]	11.89	1.41	13.79	2.03	0.44
Cattle sales price [TZS '000/sold #]	496.18	26.99	497.62	20.20	0.97
Annual cattle sales revenue [TZS '000/TLU]	171.09	19.50	183.66	39.14	0.77
Livestock productivity [$/TLU]	167.02	19.40	153.55	15.38	0.59
Farm productivity [$/ha]	2,656	725	3,143	1,070	0.71
Annual income [$/cap]	632.21	443.17	302.15	56.79	0.46
Poverty probability [PPI score]	20.66	1.34	27.15	1.54	0.00
Daily food availability [kCal/MAE]	14,848	7,413	39,477	30,027	0.43
Diet diversity [HDDS]	6.60	0.14	6.50	0.12	0.62

*TLU, tropical livestock unit (cattle herd); cap, capita; TZS '000, USD 0.43; $, Annual production or income value in international $ converted by purchasing power parity; PPI, poverty probability index; MAE, male adult equivalent; HDDS, household diet diversity score, measured for food-scarce season*.

The second descriptive comparison between farmers in the treatment and control groups includes variables assumed to be independent of ITM adoption within the timescale covered by the study, but which are expected to be associated with the dependent variables presented above. The results, presented in Table 3, show several differences between the groups. While farmers in the treatment group have larger households, a greater livestock wealth per household member and are more market-oriented, farmers in the control group have more enclosed pasture per TLU, albeit at very low levels.

**Table 3:** Descriptive statistics of independent variables for ITM treatment and control groups.

	**Control** ***n*** **=** **236**		**Treatment** ***n*** **=** **277**		
**Variable**	**Mean**	**SE**	**Mean**	**SE**	**p-value**
Household size [members]	8.54	0.40	10.45	0.40	0.00
Age of household head [years]	50.10	0.76	50.56	0.66	0.65
Education of household head (post-primary education = 1) [%]	0.30	0.03	0.23	0.03	0.11
Gender of household head (male = 1) [%]	0.89	0.02	0.93	0.02	0.10
Farm size [ha]	5.30	1.71	7.01	0.73	0.36
Enclosed pasture [ha/TLU]	0.03	0.01	0.01	0.00	0.00
Herd size [TLU/cap]	2.84	0.37	6.54	0.70	0.00
Market orientation [%]	0.17	0.02	0.22	0.02	0.06
Off-farm income share [%]	0.17	0.02	0.15	0.02	0.36

*TLU, tropical livestock unit (cattle herd); cap, capita*.

### Directly Determined ITM Effects

Based on the study's design, a set of econometric models directly investigates the links of ITM adoption with indicators at herd, farm and household level, together with a collection of other determinant variables. Table 4 presents the results regarding indicators of herd productivity. These models show positive and highly significant associations of ITM with all three productivity measures: share of calves within cattle herds, daily milk yield and annual milk sales per livestock unit.

**Table 4:** ITM vaccination and indicators of herd productivity.

	**Calf share [log(%)]**	**Daily milk yield [log(l/TLU)]**	**Annual milk sales [log(l/TLU)]**
(Intercept)	0.83 (0.70)	−0.27 (0.37)	1.47 (1.58)
ITM status (long-term = 1)	0.22^**^ (0.10)	0.20^***^ (0.06)	0.68^***^ (0.24)
Household size [log(members)]	0.18^**^ (0.08)	−0.00 (0.04)	0.47^***^ (0.18)
Age of household head [log(years)]	0.14 (0.17)	0.07 (0.09)	−0.01 (0.39)
Education of household head (post-primary = 1)	0.08 (0.10)	0.06 (0.05)	0.20 (0.22)
Gender of household head (male = 1)	−0.22 (0.14)	0.13 (0.08)	0.22 (0.33)
Herd size [log(TLU/cap)]	0.23^***^ (0.05)	−0.05^**^ (0.03)	0.36^***^ (0.11)
Enclosed pasture [ha/TLU]	−1.49^**^ (0.59)	0.26 (0.32)	0.16 (1.34)
Annual feed expenses [log(TZS ‘000/TLU)]	0.08^***^ (0.02)	0.11^***^ (0.01)	0.31^***^ (0.06)
Cattle breed type (improved = 1)	−0.19 (0.12)	0.15^**^ (0.06)	0.15 (0.27)
Market orientation [log(%)]	0.74^**^ (0.29)	1.14^***^ (0.15)	4.62^***^ (0.65)
Off-farm income share [%]	0.17 (0.15)	0.17^**^ (0.08)	0.71^**^ (0.35)
ITM status ^*^ herd size	−0.14^**^ (0.06)	−0.06^*^ (0.03)	−0.52^***^ (0.13)
ITM status ^*^ market orientation	−0.02 (0.36)	−0.58^***^ (0.20)	−1.98^**^ (0.83)
*n*	510	510	510
*R* squared	0.17	0.48	0.28
*F* statistic	7.73	35.11	14.97
*P*-value	0.00	0.00	0.00

*** *p < 0.01*;

** *p < 0.05*;

* *p < 0.1*.

*(Standard error); TLU, tropical livestock unit (cattle herd); TZS ‘000, USD 0.43; cap, capita*.

Among the other determinants, feed expenses and market orientation contribute positively to all three herd productivity indicators, while the negative interaction of ITM adoption and herd size indicates that the overall positive contribution of ITM is reduced in larger herds. The positive associations between ITM and milk yield and sales also seem to be reduced with increased market orientation, as indicated by the negative interactions. The contribution of other factors to herd productivity is more varied. Cattle herd size, measured in TLU per household member, and household size are positively associated with calf share and milk sales, while milk yields appear to be lower in larger herds. Off-farm income is positively linked to higher milk yield and sales. The type of breed is negatively linked to calf share, while the share of enclosed pasture only shows a, negative, association with calf share.

On the farm level, the econometric models include the adoption of ITM in the estimation of farm management and farm success indicators. Regarding farm management, we consider expenditure on feed, the breed type of livestock being kept and the off-take rate, calculated as sold animals per size of herd, as indicators. The results presented in Table 5 indicate no significant association of ITM vaccination with any of the three farm management indicators.

**Table 5:** ITM vaccination and indicators of farm management practices.

	**Annual feed expenses [log (TZS ‘000/TLU)]**	**Cattle breed type (improved = 1) +**	**Annual off-take [log(%)]**
(Intercept)	3.21^**^ (1.27)	3.38 (2.52)	−0.13 (0.13)
ITM status (long-term = 1)	0.31 (0.19)	0.14 (0.36)	0.03 (0.02)
Household size [log(members)]	−0.84^***^ (0.14)	−1.35^***^ (0.26)	0.02 (0.01)
Age of household head [log(years)]	0.20 (0.32)	0.00 (0.63)	0.04 (0.03)
Education of household head (post-primary = 1)	0.28 (0.18)	0.96^***^ (0.32)	−0.01 (0.02)
Gender of household head (male = 1)	−0.42 (0.26)	−0.33 (0.51)	0.03 (0.03)
Herd size [log(TLU/cap)]	−0.80^***^ (0.08)	−1.33^***^ (0.16)	0.00 (0.01)
Enclosed pasture [ha/TLU]	−0.88 (1.13)	−0.18 (2.17)	0.25^**^ (0.12)
Market orientation [log(%)]	2.36^***^ (0.51)	1.55 (0.94)	−0.05 (0.05)
Off-farm income share [%]	0.14 (0.29)	−0.14 (0.50)	0.05^*^ (0.03)
ITM status ^*^ herd size	0.27^***^ (0.10)	0.03 (0.24)	−0.03^**^ (0.01)
ITM status ^*^ market orientation	−1.61^**^ (0.66)	1.49 (1.26)	0.02 (0.07)
*n*	510	510	510
*R* squared	0.39	–	0.04
*F* statistic	28.48	–	2.00
*P*-value	0.00	–	0.03

*** *p < 0.01*;

** *p < 0.05*;

* *p < 0.1*.

*(Standard error); + Logit model: Pseudo R Squared = 0.482, Prob Chi^2^ < 0.001; TLU, tropical livestock unit (cattle herd); TZS ‘000, USD 0.43; cap, capita*.

Of the other factors associated with improved farm management practices there seem to be more similarities in the determinants of feed expenses and breed type compared to off-take rate. Herd and household sizes show negative associations with the two former indicators, while market orientation has a positive coefficient only for feed expenses. In contrast, the off-take rate appears to be mainly linked to the share of enclosed pasture and of off-farm income.

The models investigating ITM adoption and farm success, shown in Table 6, include as dependent variables the average sales price and the average annual sales revenue of cattle, as well as livestock and farm productivity. Here, ITM adoption shows a significant contribution to livestock productivity only.

**Table 6:** ITM vaccination and indicators of farm success.

	**Cattle sales price [log(TZS ‘000/sold #)]**	**Annual cattle sales revenue [log(TZS ‘000/TLU)]**	**Livestock productivity [log($/TLU)]**	**Farm productivity [log($/ha)]**
(Intercept)	5.59^***^ (0.58)	4.11^***^ (1.13)	2.25^*^ (1.22)	8.56^***^ (1.19)
ITM status (long-term = 1)	0.13 (0.09)	0.17 (0.17)	0.78^***^ (0.18)	0.17 (0.18)
Household size [log(members)]	−0.11^*^ (0.06)	−0.14 (0.11)	0.25^*^ (0.13)	0.30^**^ (0.13)
Age of household head [log(years)]	0.20 (0.14)	0.28 (0.28)	0.00 (0.31)	−0.74^**^ (0.30)
Education of household head (post-primary = 1)	0.06 (0.08)	0.14 (0.15)	0.04 (0.17)	−0.07 (0.16)
Gender of household head (male = 1)	−0.11 (0.14)	−0.09 (0.27)	−0.01 (0.26)	0.03 (0.26)
Herd size [log(TLU/cap)]	−0.07 ^*^ (0.04)	−0.52^***^ (0.08)	−0.06 (0.07)	−0.04 (0.07)
Enclosed pasture [ha/TLU]	0.25 (0.45)	2.37^***^ (0.86)	0.15 (1.00)	−1.22 (0.95)
Market orientation [log(%)]	0.58^**^ (0.25)	0.38 (0.49)	5.61^***^ (0.49)	1.97^***^ (0.47)
Off-farm income share [%]	−0.03 (0.12)	−0.17 (0.23)	0.38 (0.27)	0.41 (0.26)
ITM status ^*^ herd size	0.02 (0.05)	−0.01 (0.09)	−0.36^***^ (0.10)	0.06 (0.10)
ITM status ^*^ market orientation	−0.38 (0.31)	−0.06 (0.59)	−2.11^***^ (0.63)	−0.72 (0.61)
*n*	275	275	507	486
*R* squared	0.09	0.42	0.37	0.08
*F* statistic	2.47	17.16	26.56	3.91
*P*-value	0.01	0.00	0.00	0.00

*** *p < 0.01*;

** *p < 0.05*;

* *p < 0.1*.

*(Standard error); TLU, tropical livestock unit (cattle herd); TZS ‘000, USD 0.43; cap, per capita; $, Annual production value in international $ converted by purchasing power parity*.

Among the other determinants included in these models, market orientation and household size are linked to the productivity indicators. The positive association of ITM with livestock productivity appears to be reduced by both herd size and market orientation. Sales revenue per animal being kept is reduced by herd size but increased by enclosed pasture. The cattle price appears to be mainly associated with market orientation.

Finally, the results of the direct association of ITM adoption with the livelihood indicators considered in this study, namely gross per capita income, poverty probability, food availability and household diet diversity, are shown in Table 7. The adoption of ITM shows significant contributions to all indicators with the expected signs; the negative sign for poverty probability indicates a reduced poverty risk. However, the positive associations of ITM, herd size and market orientation with livelihood indicators appear to be weaker when combined, as shown by their negative interaction terms in most cases.

**Table 7:** ITM vaccination and household livelihood indicators.

	**Annual income [log($/cap)]**	**Poverty probability [log (PPI score)]**	**Daily food availability [log (kCal/MAE)]**	**Diet diversity [log(HDDS)]**
(Intercept)	2.31^**^ (1.01)	4.41^***^ (0.76)	8.46^***^ (1.04)	1.66^***^ (0.25)
ITM status (long-term = 1)	0.46^***^ (0.15)	−0.20^*^ (0.12)	0.61^***^ (0.16)	0.09^**^ (0.04)
Household size [log(members)]	0.21^*^ (0.11)	0.74^***^ (0.09)	−0.17 (0.12)	−0.07^**^ (0.03)
Age of household head [log(years)]	−0.61^**^ (0.26)	−0.72^***^ (0.19)	−0.26 (0.26)	0.10 (0.06)
Education of household head (post-primary = 1)	−0.10 (0.14)	−0.72^***^ (0.11)	0.06 (0.14)	0.10^***^ (0.03)
Gender of household head (male = 1)	0.06 (0.22)	−0.11 (0.17)	0.16 (0.23)	−0.07 (0.05)
Herd size [log(TLU/cap)]	0.13^**^ (0.06)	−0.00 (0.05)	0.33^***^ (0.06)	0.06^***^ (0.02)
Enclosed pasture [ha/TLU]	0.89 (0.87)	−0.02 (0.65)	−0.29 (0.89)	0.30 (0.22)
Market orientation [log(%)]	10.83^***^ (0.41)	−1.27^***^ (0.31)	2.99^***^ (0.42)	0.32^***^ (0.10)
Off-farm income share [%]	1.57^***^ (0.23)	−0.31^*^ (0.17)	0.78^***^ (0.23)	0.15^***^ (0.06)
ITM status ^*^ herd size	−0.06 (0.08)	0.15^**^ (0.06)	−0.19^**^ (0.09)	−0.08^***^ (0.02)
ITM status ^*^ market orientation	−1.64^***^ (0.53)	0.65 (0.40)	−1.56^***^ (0.55)	−0.31^**^ (0.13)
*n*	507	507	507	507
*R* squared	0.77	0.36	0.23	0.14
*F* statistic	147.07	25.49	13.41	7.21
*P*-value	0.00	0.00	0.00	0.00

*** *p < 0.01*;

** *p < 0.05*;

* *p < 0.1*.

*(Standard error); TLU, tropical livestock unit (cattle herd); cap, per capita; $, Annual income value in international $ converted by purchasing power parity; PPI, poverty probability index; MAE, male adult equivalent; HDDS, household diet diversity score, measured for food scarce season*.

Only market integration and off-farm income show strong positive contributions across all four livelihood indicators. Herd size is also positively linked to all livelihood indicators except for poverty probability. However, this indicator shows significant coefficients for household size, as well as age and education of household head, the latter negatively. Education is also associated with household diet diversity, though positively.

### Effects of Intermediate Outcomes

In addition to considering ITM adoption directly at various levels of analysis, this study also attempts to follow the indirect outcomes linked to this technology along the pathways outlined in the conceptual framework illustrated above. For this, the outcome indicators at one level are included as determinants, i.e., independent variables, at the next level, instead of the ITM adoption variable. For consistency, these have been considered irrespective of whether they were significantly associated with ITM adoption or not.

The first such set of models investigates the contribution of herd productivity outcomes to farm management indicators. The results, presented in Table 8, show mixed associations. Milk yield and sales are closely associated with feed expenses and breed type, although the causality and the negative sign for milk sales remain to be discussed. Calf share only shows a negative association with breed type, indicating a higher calf share in herds with more local breeds. The model predicting off-take rate does not appear to be significant.

**Table 8:** Association of herd productivity with farm management indicators.

	**Annual feed expenses [log (TZS ‘000/TLU)]**	**Cattle breed type (improved = 1) +**	**Annual off-take [log(%)]**
(Intercept)	3.31^***^ (1.19)	4.49^*^ (2.67)	−0.10 (0.13)
Calf share [log(%)]	0.09 (0.08)	−0.38^*^ (0.20)	−0.00 (0.01)
Daily milk yield [log(l/TLU)]	1.80^***^ (0.24)	2.65^***^ (0.59)	0.02 (0.03)
Annual milk sales [log(TZS ‘000/TLU)]	−0.20^***^ (0.06)	−0.36^***^ (0.12)	−0.01 (0.01)
Household size [log(members)]	−0.58^***^ (0.13)	−1.06^***^ (0.27)	0.02 (0.01)
Age of household head [log(years)]	0.03 (0.30)	−0.18 (0.66)	0.04 (0.03)
Education of household head (post-primary = 1)	0.08 (0.16)	0.75^**^ (0.33)	0.00 (0.02)
Gender of household head (male = 1)	−0.54^**^ (0.25)	−0.40 (0.52)	0.03 (0.03)
Herd size [log(TLU/cap)]	−0.37^***^ (0.06)	−0.93^***^ (0.13)	−0.01 (0.01)
Enclosed pasture [ha/TLU]	−0.67 (1.05)	−1.34 (2.36)	0.20^*^ (0.12)
Market orientation [log(%)]	0.27 (0.34)	1.90^***^ (0.69)	0.01 (0.04)
Off-farm income share [%]	−0.04 (0.27)	−0.36 (0.54)	0.06^*^ (0.03)
*n*	510	510	510
*R* squared	0.47	–	0.04
*F* statistic	39.54	–	1.67
*P*-value	0.00	–	0.08

*** *p < 0.01*;

** *p < 0.05*;

* *p < 0.1*.

*(Standard error); + Logit model: Pseudo R Squared = 0.517, Prob Chi^2^ < 0.001; TLU, tropical livestock unit (cattle herd); TZS ‘000, USD 0.43; cap, capita*.

Beyond the herd productivity outcomes, household and herd size show negative links with feed and breed indicators in these models, but there is a positive contribution by market orientation to breed type. Also, female household heads appear to invest more in feeds while more educated household heads favor improved cattle breeds.

On the next level, the contribution of the farm management indicators to farm success are shown in Table 9. Here, feed expenses and off-take rate have significant positive links with livestock productivity. While off-take is also linked to sales revenue, breed type is, unsurprisingly, associated with higher cattle prices.

**Table 9:** Association of farm management with farm success indicators.

	**Cattle sales price [log(TZS ‘000/sold #)]**	**Annual cattle sales revenue [log(TZS ‘000/TLU)]**	**Livestock productivity [log($/TLU)]**	**Farm productivity [log($/ha)]**
(Intercept)	5.72^***^ (0.59)	3.38^***^ (0.86)	2.00 (1.24)	8.17^***^ (1.20)
Annual feed expenses [log(TZS ‘000/TLU)]	−0.01 (0.02)	0.03 (0.03)	0.15^***^ (0.04)	0.03 (0.04)
Cattle breed type (improved = 1)	0.19^**^ (0.09)	0.16 (0.12)	−0.01 (0.21)	0.33^*^ (0.20)
Annual off-take [log(%)]	−0.10 (0.19)	3.84^***^ (0.27)	0.95^**^ (0.40)	−0.76^**^ (0.38)
Household size [log(members)]	−0.07 (0.06)	0.05 (0.09)	0.37^**^ (0.14)	0.42^***^ (0.14)
Age of household head [log(years)]	0.15 (0.14)	0.12 (0.21)	−0.03 (0.31)	−0.73^**^ (0.30)
Education of household head (post-primary = 1)	0.02 (0.08)	−0.03 (0.12)	0.09 (0.17)	−0.13 (0.16)
Gender of household head (male = 1)	−0.11 (0.14)	−0.28 (0.21)	−0.03 (0.26)	0.06 (0.25)
Herd size [log(TLU/cap)]	−0.03 (0.03)	−0.15^***^ (0.05)	−0.11^*^ (0.07)	0.08 (0.06)
Enclosed pasture [ha/TLU]	0.14 (0.44)	0.95 (0.64)	−0.21 (1.00)	−1.07 (0.94)
Market orientation [log(%)]	0.30^**^ (0.15)	0.35 (0.21)	4.37^***^ (0.33)	1.35^***^ (0.31)
Off-farm income share [%]	−0.03 (0.12)	−0.05 (0.17)	0.31 (0.27)	0.45^*^ (0.26)
*n*	275	275	507	486
*R* squared	0.10	0.67	0.36	0.10
*F* statistic	2.59	48.93	25.09	4.63
*P*-value	0.00	0.00	0.00	0.00

*** *p < 0.01*;

** *p < 0.05*;

* *p < 0.1*.

*(Standard error); TLU, tropical livestock unit (cattle herd); TZS ‘000, USD 0.43; cap, capita; $, Annual production value in international $ converted by purchasing power parity*.

Household size and market orientation are significantly linked to increased livestock and farm productivity in these models. Smaller herds seem to imply higher average sales revenues and livestock productivity. Once again, off-farm income seems unrelated to farm success, except for farm productivity.

Finally, Table 10 presents the contributions of farm success outcomes on livelihood indicators. Both livestock and farm productivity are strongly linked to increases in income and food availability. Cattle sales revenue, unsurprisingly, increases income, but is not linked to the other livelihood indicators, while the cattle price does not seem to show any significant associations. Diet diversity is not associated with any of the farm success indicators.

**Table 10:** Association of farm success with household livelihood indicators.

	**Annual income [log($/cap)]**	**Poverty probability [log (PPI score)]**	**Daily food availability [log (kCal/MAE)]**	**Diet diversity [log(HDDS)]**
(Intercept)	−3.89^**^ (1.59)	8.01^***^ (1.34)	3.51^***^ (1.31)	1.80^***^ (0.40)
Cattle sales price [log(TZS '000/sold #)]	−0.01 (0.17)	−0.18 (0.14)	0.04 (0.14)	−0.04 (0.04)
Annual cattle sales revenue [log(TZS '000/TLU)]	0.22^**^ (0.09)	0.06 (0.07)	0.06 (0.07)	0.00 (0.02)
Livestock productivity [log($/TLU)]	0.34^***^ (0.06)	−0.08 (0.05)	0.23^***^ (0.05)	0.02 (0.02)
Farm productivity [log($/ha)]	0.23^***^ (0.06)	−0.09^*^ (0.05)	0.43^***^ (0.05)	0.01 (0.01)
Household size [log(members)]	0.15 (0.13)	0.76^***^ (0.11)	−0.35^***^ (0.11)	−0.09^***^ (0.03)
Age of household head [log(years)]	0.05 (0.34)	−1.21^***^ (0.29)	0.06 (0.28)	0.11 (0.09)
Education of household head (post-primary = 1)	−0.10 (0.18)	−0.69^***^ (0.15)	0.21 (0.15)	0.10^**^ (0.05)
Gender of household head (male = 1)	0.26 (0.32)	−0.19 (0.27)	0.39 (0.26)	−0.07 (0.08)
Herd size [log(TLU/cap)]	0.46^***^ (0.07)	0.09 (0.06)	0.42^***^ (0.06)	0.01 (0.02)
Enclosed pasture [ha/TLU]	0.63 (1.01)	−0.61 (0.84)	0.39 (0.82)	−0.12 (0.25)
Market orientation [log(%)]	8.97^***^ (0.40)	−0.74^**^ (0.34)	0.73^**^ (0.33)	0.06 (0.10)
Off-farm income share [%]	1.46^***^ (0.28)	−0.57^**^ (0.23)	0.44^*^ (0.23)	0.20^***^ (0.07)
*n*	261	261	261	261
*R* squared	0.82	0.43	0.59	0.16
*F* statistic	91.84	15.83	29.25	3.99
*P*-value	0.00	0.00	0.00	0.00

*** *p < 0.01*;

** *p < 0.05*;

* *p < 0.1*.

*(Standard error); TLU, tropical livestock unit (cattle herd); TZS ‘000, USD 0.43; cap, capita; $, Annual income value in international $ converted by purchasing power parity; PPI, poverty probability index; MAE, male adult equivalent; HDDS, household diet diversity score, measured for food scarce season*.

Off-farm income contributes significantly to all four livelihood indicators, positively. The only other significant determinants of diet diversity are smaller households and higher education. Market orientation is linked to improved income, poverty risk and food availability, while herd size is associated positively with income and food availability. Poverty probability is also decreased by smaller household size as well as by higher age and education of the household head.

## Discussion

In contrast to most studies on the ITM vaccine, which focus on restricted areas and on interventions targeted at specific communities or production systems, this study aims to provide representative insights into outcomes associated with ITM adoption within major cattle-keeping areas of Tanzania and where ITM has been promoted for many years. On the other hand, ITM adoption is not yet ubiquitous, which would have made the identification of a control group as a counterfactual very difficult. Therefore, the stage of the scaling process at which this study was implemented appears to have been appropriate. Nevertheless, finding enough eligible and collaborative animal health service providers and generating a sufficiently large and accurate sampling frame of farmers presented a challenge, especially when attempting to consider multiple distinct production systems, generally mentioned as a major characteristic when describing the Tanzanian livestock sector (29). However, because a simple categorization of systems, for instance into dairy and pastoralist farmers, could neither be achieved in the sampling frame nor in the collected data, this aspect was not considered. According to variables recording herd mobility, only very few respondents would have been characterized as pastoralists. Discussions with stakeholders suggested that the concept of pastoralism was sensitive at the time of the survey, with many administrative efforts aimed at restricting the movement of livestock. This is in line with other findings showing that livestock production systems in Tanzania are becoming less distinct with many pastoralists engaging in crop production and reducing their transhumance (30). Therefore, it appears justified not to consider this aspect explicitly in the current analysis, especially as production system characteristics, such as herd size, cultivated land or enclosed grazing area, are already included. Nevertheless, a better understanding of current production systems and their linkages with animal health management would be useful when investigating technology adoption patterns and their determinants. However, these research objectives are not considered in this study and would require further and larger investigations. Also, the fact that the collected data could not accurately record animal health practices, especially regarding the control of ticks and the treatment of ECF, is a limitation of this study. It is often difficult to record individual, irregular farm management activities over a 12-month recall period, especially if they are associated with illness and death of animals. However, following the number of livestock keepers included in this study regularly throughout a 12-month period to record such data with shorter recall periods, for instance 30 days, would require substantially greater resources. The sampling approach for this study appears appropriate, although, had it been possible to identify more “just-starting” farmers, the number of additions from the group of non-adopters selected by propensity score matching would have been reduced. And a larger overall sample would have been better able to determine effects on variables with small differences between analysis groups, such as the cattle price. Finally, only a panel survey with a randomized application of the ITM vaccine would be able to overcome the uncertainty whether earlier adopters, defined here as “long-term” and considered as the treatment group, were not statistically different at the time of their ITM adoption to the more recent adopters, included in the study as “just-starting” and as the counter-factual. These differences between adopter types, building on Rogers' Diffusion of Innovation theory (31), could, for instance, apply to their risk behavior, innovation capacity, production intensity or livelihood indicators. However, even the “long-term” adopters in this sample might not represent typical early adopters in the sense of the innovation diffusion theory as most of them adopted ITM only 3 years prior to the survey while ITM had been available for nearly 30 years. Another bias could have occurred if the two groups had differed considerably in their production systems, even though the study could not effectively determine this. This would have been relevant, if, for instance, ITM had been targeted at some production systems earlier than others. The farm descriptives in Table 3 do indicate some differences between groups, for instance in herd size and enclosed pasture. However, with farm size being indistinguishable, the two groups don't seem to differ considerably regarding their production system composition. Nevertheless, a credible counterfactual remains the basis for assuming causal relationships. Therefore, we refrain from interpreting the associations of ITM as impacts, which would imply causality. However, we are convinced that the various significant associations between the adoption of ITM and several relevant indicators provide valuable insights into the assessment of this important technology.

The results of the econometric models constructed according to the conceptual framework along hypothesized impact pathways vary considerable by the level of investigation. The adoption of the ITM vaccine is associated significantly and positively with the three herd productivity indicators. This is reassuring as these are the basis of most of the expected further benefits of ITM, apart from the reductions in the cost of tick control and ECF treatment. However, this study, with its focus on farm and household data, cannot determine the actual causes of the associations with milk yield and sales; whether they, for instance, result from more calves being available for stimulating lactation or because of improved cow health. Both causal links have been suggested. Nevertheless, the positive associations with ITM confirm various ex-ante and ex-post studies indicating the vaccine's benefits (3, 24). While feed expenses were associated significantly with all three herd productivity indicators, breed type appeared to be linked only to milk yield. The negative ITM—herd size interaction with all three indicators suggests that the productivity effect of ITM is higher in smaller herds. In general, it is assumed that smaller herds are more oriented toward dairy production and markets while larger herds resemble more extensive pastoral production systems. Changes in productivity might be easier and faster to determine, where milk is the main product and links to markets are stronger. This negative interaction contrasts with the general perception that farmers with larger herds are more eager to engage with vaccinators, partly because of the greater efficiency when vaccinating many animals at once. While the results indicate that ITM adoption offers considerable potential for improving reproduction and milk production in small herds, a more focused analysis would be necessary to comparatively determine the effects of ITM adoption in small and large herds and whether ITM might even lead to negative effects in some large herds.

However, the hypothesized changes to farm management practices, such as increased feed expenses, switching to improved breeds or a higher off-take rate, could not be determined. While the previous results had shown that more feed purchases increase herd productivity, the longer-term reverse causal link of ITM vaccination, representing a higher production potential, stimulating a greater use of feed inputs could not be detected. This also applies to the off-take rate and the share of herds with improved breeds, despite previous findings on changes in breed composition by ITM adopters (18). In addition, clearly differentiating animals of local breeds, which are often genetically mixed, from improved cross-bred animals is challenging in a survey situation. The included household characteristics, such as size, education and gender of head as well as market orientation appear to have greater influence on production intensification than ITM adoption within the period covered by this study.

On the other hand, ITM adoption does seem to have a positive link with livestock productivity, while the other measures of farm success appear to be unrelated. Any increases in cattle sales appear to be driven mainly by the off-take rate, which does not seem to be linked to ITM vaccination. That cattle prices are also not increased by ITM vaccination contrasts with various reports that ear-tags on marketed livestock indicating ITM vaccination result in a price premium. Among the other factors determining farm success, market orientation contributes most strongly, which is not surprising as farm success is mainly defined in market terms. Interestingly, average cattle sales revenue is not significantly associated with market orientation, as is the case with the off-take rate, which could be another indication of the importance of non-market production objectives in beef-oriented systems. That female-headed households appear to do better regarding cattle prices and farm productivity might warrant further investigation, but this is beyond the scope of this study.

Finally, the cumulative nature of benefits resulting from a specific intervention such as a livestock vaccine are highlighted in the positive effects of ITM adoption in all four livelihood-oriented models. This was not necessarily to be expected, as various other determinants might mask the influence of ITM on the livelihood indicators while moving through the levels of investigation from herd to household level. Nevertheless, it appears that the effects of ITM adoption on livestock productivity—and the importance of livestock—were strong enough to be significantly associated with livelihood improvements of the interviewed ITM adopters. However, it appears that the strength of ITM's benefits varies by type of farm household. For instance, farms with larger herds seem to see less improvements in food availability and diet diversity when adopting ITM, compared to those with smaller herds as shown by the significant interaction terms. A similar effect is seen regarding market orientation. This underlines the need for gaining a better understanding of how the impact pathways of ITM adoption differ amongst various types of livestock keepers and whether adopting ITM might even lead to detrimental effects of some farmers. These insights would also be relevant for investigating patterns of ITM adoption.

An alternative approach to studying the separate steps along the impact pathway in greater detail is to include the dependent variables of one level as independent variables at the next level. The results from assessing the association of herd-level indicators on farm management confirm the earlier findings in this study that improved herd productivity does not appear to support overall production intensification within the study's observation period. Rather, specific productivity outcomes show contrasting effects. For instance, increased calf share shows a negative association with breed type. Raising calves beyond replacement requirements might be unattractive in dairy systems, which is where improved breeds have mainly been introduced. On the other hand, higher milk yield is associated with production intensification. It is however challenging to interpret the negative coefficient of milk sales with feed expenses and breed type. Whether home consumption plays a sufficiently important role to explain the difference between yield and sales or whether a correlation with other determinants is at play is difficult to determine within this study.

At the next level, the beef-oriented pathway seems to again show the strongest linkage, with off-take rate associated with both cattle sales and overall livestock productivity, while feed expenses are only linked to livestock productivity increases. However, it must be acknowledged that potential direct contributions to livestock productivity by herd-level outcomes such as milk production and sales are not considered in these models. The negative association of off-take with whole farm productivity, which includes crop production and is calculated per farm area, is again most likely due to differences in farm characteristics and production orientation. Finally, out of the included farm success indicators both livestock and farm productivity significantly improve income and food availability. Diet diversity seems to be dominated by the availability of off-farm income in this model.

While these results confirm the beneficial effects of adopting ITM amongst a wide range of livestock farmers in Tanzania, questions remain regarding differences in magnitude and impact pathways amongst different farm households. It would be especially interesting to study the differences more intensively between dairy- and beef-oriented producers regarding the interlinked determinants of farm success and livelihood improvements. It appears that while the conceptual framework introduced in this study is useful for a basic understanding of potential impact pathways it does not sufficiently capture effects across several levels and within levels. For instance, the effect of milk sales on livestock productivity is obscured by the intermediate farm management practices. Also, it could be argued that food security is more directly dependent on income or poverty indicators rather than on farm success, implying the importance of impact links within the levels defined for this study. It would need further discussion to determine whether the greater clarity gained by grouping indicators into levels outweighs the drawbacks of missing out on perhaps crucial linkages within these levels. However, variables within the same levels might also be highly correlated, which would challenge the interpretation of results if included in the same model.

Despite the difficulties in identifying and quantifying the most relevant impact pathways within these farm households, this approach appears essential to gain a better understanding of how the ITM vaccination—and other interventions aimed at improving livestock productivity—cause livelihood improvements. Only then will it be possible to efficiently target dissemination activities, create supportive conditions and anticipate the effects of overall development trends.

## Data Availability Statement

The raw data supporting the conclusions of this article will be made available by the authors, without undue reservation.

## Ethics Statement

The studies involving human participants were reviewed and approved by Institutional Research Ethics Committee (IREC) International Livestock Research Institute (ILRI). The patients/participants provided their written informed consent to participate in this study.

## Author Contributions

NT led the design and implementation of the study as well as the development of the analysis and of the manuscript. LK supervised the survey implementation, implemented the analysis, and contributed to the manuscript. JH, MW, and HK contributed to the design of the study and the data collection tool as well as to the manuscript. All authors contributed to the article and approved the submitted version.

## Funding

Funding for this study was provided through the CGIAR Research Programme on Livestock (https://livestock.cgiar.org/). We thank all partners and all donors that globally support our work through their contributions to the CGIAR system (http://www.cgiar.org/about-us/our-funders).

## Conflict of Interest

The authors declare that the research was conducted in the absence of any commercial or financial relationships that could be construed as a potential conflict of interest.

## Publisher's Note

All claims expressed in this article are solely those of the authors and do not necessarily represent those of their affiliated organizations, or those of the publisher, the editors and the reviewers. Any product that may be evaluated in this article, or claim that may be made by its manufacturer, is not guaranteed or endorsed by the publisher.
